# Microvascular ultrasound imaging of the neonatal brain: a scoping review

**DOI:** 10.1007/s00431-026-07042-x

**Published:** 2026-05-21

**Authors:** Anoop Ramana, Jingwen Zhu, Ksenija Acimovic, Kalyan Mitra, Meng-Xing Tang, Jayanta Banerjee

**Affiliations:** 1 Neonatal Intensive Care Unit, Queen Charlotte’s and Chelsea Hospital, Imperial College Healthcare NHS Trust, London, W12 0HS UK; 2 Ultrasound Laboratory for Imaging and Sensing Group, Department of Bioengineering, Imperial College London, London, SW7 2AZ UK; 3 Department of Neonatology, Primary Care Public Health, Faculty of Medicine, Imperial College London, London, SW7 2AZ UK; 4 Centre for Paediatrics and Child Health, Origins of Child Health and Disease, Imperial College London, London, SW7 2AZ UK

**Keywords:** Microvascular, Neuroimaging, Neonatal brain, Super-resolution ultrasound, Contrast-enhanced ultrasound, Ultrafast Doppler imaging

## Abstract

Microvascular changes in the developing neonatal brain often precede clinically detectable injury. Advanced ultrasound techniques such as microvascular Doppler (MVD), contrast-enhanced ultrasound (CEUS), and super-resolution ultrasound (SRUS) offer the potential for bedside assessment of microvascular flow and perfusion without sedation, organotoxic contrast agents, or ionising radiation. Using Joanna Briggs Institute methodology and PRISMA-ScR guidance, MEDLINE, EMBASE, Web of Science, trial registries, and grey literature were searched for original in vivo neonatal studies reporting microvascular or perfusion data using MVD, CEUS, or SRUS-based approaches. Extracted data included study metadata, population characteristics, ultrasound systems and protocols, and key feasibility, safety, and performance outcomes. Of 3028 screened records, 23 studies met the inclusion criteria (1 SRUS, 8 CEUS, 14 MVD). Using SonoVue/Lumason microbubbles, the CEUS studies reported 163 neonatal brain scans in 91 neonates (25^+3^ to 41^+4^ weeks’ gestation). A total of 263 infants were studied across 14 MVD studies. All studies demonstrated safe ultrasound techniques that can be used to assess brain perfusion and cerebrovascular pathologies in preterm and term neonates, particularly in understanding changes in cerebral microvasculature, mainly in infants with hypoxic–ischaemic encephalopathy (HIE), neurovascular, and congenital cardiac conditions.

*Conclusions*: Advanced ultrasound techniques show promise as adjuncts in neonatal neuroimaging, enabling assessment of perfusion and microvascular haemodynamics. Future clinical translation would require standardised protocols, normative datasets, longitudinal outcome linkage, governance and training standards, and diagnostic effectiveness.

**Supplementary Information:**

The online version contains supplementary material available at 10.1007/s00431-026-07042-x.

**Table:** 

**What is Known:** • *Conventional cranial ultrasound is indispensable in neonatal care but predominantly demonstrates anatomical structures. Doppler ultrasound provides macrovascular surrogates and does not directly quantify tissue perfusion*. • *Microvascular dysfunction is a fundamental antecedent to most neonatal brain injuries yet remains largely undetectable to current bedside imaging*.		
**What is New:** • *This scoping review synthesises in vivo neonatal evidence for microvascular ultrasound techniques for bedside assessment*. • *Evidence to date supports feasibility across diverse neonatal groups and thus readiness for mechanistic evaluation in health and disease*. • *Translational progress is limited by small cohorts, a lack of normative reference datasets, and limited linkage with longitudinal outcomes*.		

## Introduction

Neonatal brain injury refers to injury to the developing brain occurring during the perinatal period, encompassing conditions that disrupt cerebral blood flow, oxygenation, microvascular integrity, or parenchymal development [1]. In England, newborn brain injury affects approximately 1 in 250 infants, with markedly higher rates amongst preterm infants and substantial long-term neurocognitive morbidity. In the UK, neonatal mortality surveillance reports continue to identify brain injury syndromes such as GMH/IVH and HIE as major contributors to neonatal deaths [2]. Survivors can develop cerebral palsy, epilepsy, sensory impairment, and cognitive disability, with profound impacts on families and healthcare systems. The physiological objective of many neonatal neuroprotective strategies is to reduce sudden perturbations in cerebral blood flow and limit secondary injury. Although the timing of injury is often uncertain, a significant proportion occurs within the first few days of life [3, 4]. Diagnosis relies heavily on serial bedside cranial ultrasonography, which provides predominantly anatomical information and delineates major intra-ventricular bleeds [5]. Conventional Doppler adds haemodynamic context but is limited to angle-dependent macrovascular flow surrogates and does not directly assess tissue perfusion [6]. Low spatial resolution means microvascular structures remain below the detection threshold. MRI provides superior tissue characterisation but is commonly performed later (often at term-corrected age in preterm infants), when injury may already be established, and can be limited by access, cost, transport, and clinical stability [7, 8].

### Why are microvascular-level haemodynamic tools needed in neonatology?

The neonatal neuroimaging community has long sought bedside haemodynamic markers that meaningfully predict injury and outcome. A recent systematic review of cerebral Doppler in preterm infants provides an important reference point for the limitations of the current paradigm [6]. Across 23 included studies and substantial heterogeneity in technique, vessels, timing, and outcomes, there was no clear evidence supporting the routine use of arterial Doppler-derived indices (including RI and related parameters) to predict neurodevelopmental outcome. The review identified important methodological constraints: angle dependence for velocity-based measures, inconsistent correction for confounders, limited blinding, and heterogeneous reporting that prevented quantitative synthesis. Even where associations were described (e.g. elevated RI in ACA/MCA in the context of haemodynamically significant PDA and low RI in HIE), these findings did not translate into robust predictive tools for brain injury or long-term neurodevelopment. Recent scoping reviews of infant cerebral arterial and venous Doppler waveforms highlight both the clinical value of Doppler-based haemodynamic assessment and its main limitation: most studies characterise waveform changes in larger vessels, rather than directly imaging tissue-level perfusion or capillary-scale microvascular structure [9, 10].

These limitations are not simply statistical and reflect a biological mismatch between conventional Doppler measures and what clinicians need. Macrovascular velocity surrogates are indirect and may be dominated by systemic shunts, vascular tone, ventilatory strategies, and insonation geometry. In contrast, many neonatal brain injuries emerge from microvascular instability: impaired autoregulation, venous congestion, capillary transit heterogeneity, oxygen diffusion failure, and neuroinflammation-driven microcirculatory dysfunction. These processes can precede or occur without early macrovascular changes detectable by routine Doppler, making a strong case for tools that directly interrogate tissue perfusion and microvascular architecture rather than relying on upstream surrogates.

Microvascular dysfunction and impaired autoregulation underpin many types of neonatal brain injury but remain largely invisible to current neuroimaging techniques. Neonatal intensive care is rich in monitoring technology, yet few devices have been designed to interrogate neonatal cerebral microvascular physiology directly at the bedside. Advanced haemodynamic and microvascular ultrasound approaches have emerged and can be broadly divided into microvascular Doppler (MVD), contrast-enhanced ultrasound (CEUS), and super-resolution ultrasound (SRUS) [11–16]. MVD encompasses Doppler-derived techniques that visualise slow flow within small vessels, typically in the millimetre to hundreds-of-microns range, most commonly without contrast agents. CEUS employs intravenously administered microbubble contrast agents to enhance the detection of capillary-level blood flow in real time, enabling a direct and dynamic assessment of tissue perfusion. CEUS is already well established in adult radiology for bedside characterisation of focal liver lesions such as hepato-cellular carcinoma and haemangioma based on perfusion patterns. SRUS goes beyond the fundamental diffraction limit that constrains the spatial resolution of conventional ultrasound, enabling microvascular structures to be both detected and spatially resolved. Ultrasound localisation microscopy (ULM) represents one implementation of this approach by localising and tracking individual microbubbles [17, 18]. These approaches, therefore, can be seen as complementary to current neonatal neuroimaging, depending on the clinical application.

This commissioned review summarises the available evidence for microvascular ultrasound imaging in the neonatal brain. For clinical relevance and translation, the focus is on feasibility, safety, clinical applicability, and key existing knowledge gaps.

## Methods

This scoping review is reported using the Preferred Reporting Items for Systematic Reviews and Meta-Analyses for Scoping Review (PRISMA-ScR) checklist [19]. An initial search was performed to identify key articles most relevant to the topic. These results were inserted into the Yale MeSH analyser to generate initial search terms. Next, the Patient, Concept, and Context (PCC) framework was used to develop the research questions and inclusion criteria [20]. A final search strategy was created that directly aligned with the research objectives: Population: neonatal human participants of any age or sex; Concept: any use of ultrasound for microvascular imaging and perfusion assessment; and Context: original research articles from 1946 to 2025 studying the neonatal brain. The results were applied to advanced searches in MEDLINE, EMBASE, and Web of Science databases from 1946 to 2025 to identify relevant studies in the English language [20, 21]. All search results were imported and de-duplicated using Covidence (Veritas Health Innovation, Melbourne, Australia) [22]. The screening process involved two independent reviewers for initial review, and findings were compared collaboratively. Discrepancies or inconsistencies were resolved through discussions with the supervising author. Publications with primary data on the use of ultrasound in neonatal brain microvascular imaging were included, and data regarding population, technological materials, context, and outcomes pertaining to applicability, tolerability, and repeatability of the studies were extracted. A total of 25 standardised data items were chosen and extracted for all included articles to reflect our review questions (Supplementary Table 1) [23].

The primary question was: What are the key findings from the literature on the use of ultrasound in neonatal brain microvascular and perfusion assessment, its place in routine neonatal intensive care, its benefits, safety, and the challenges ahead? Secondary questions included: (a) What ultrasound systems and materials were used? (b) What relevant outcome measures are used, and what information is relevant to clinicians? (c) What key findings were reported, aligning with our PCC framework? (d) What is the current state of clinical integration?

## Results

**Fig. 1:**
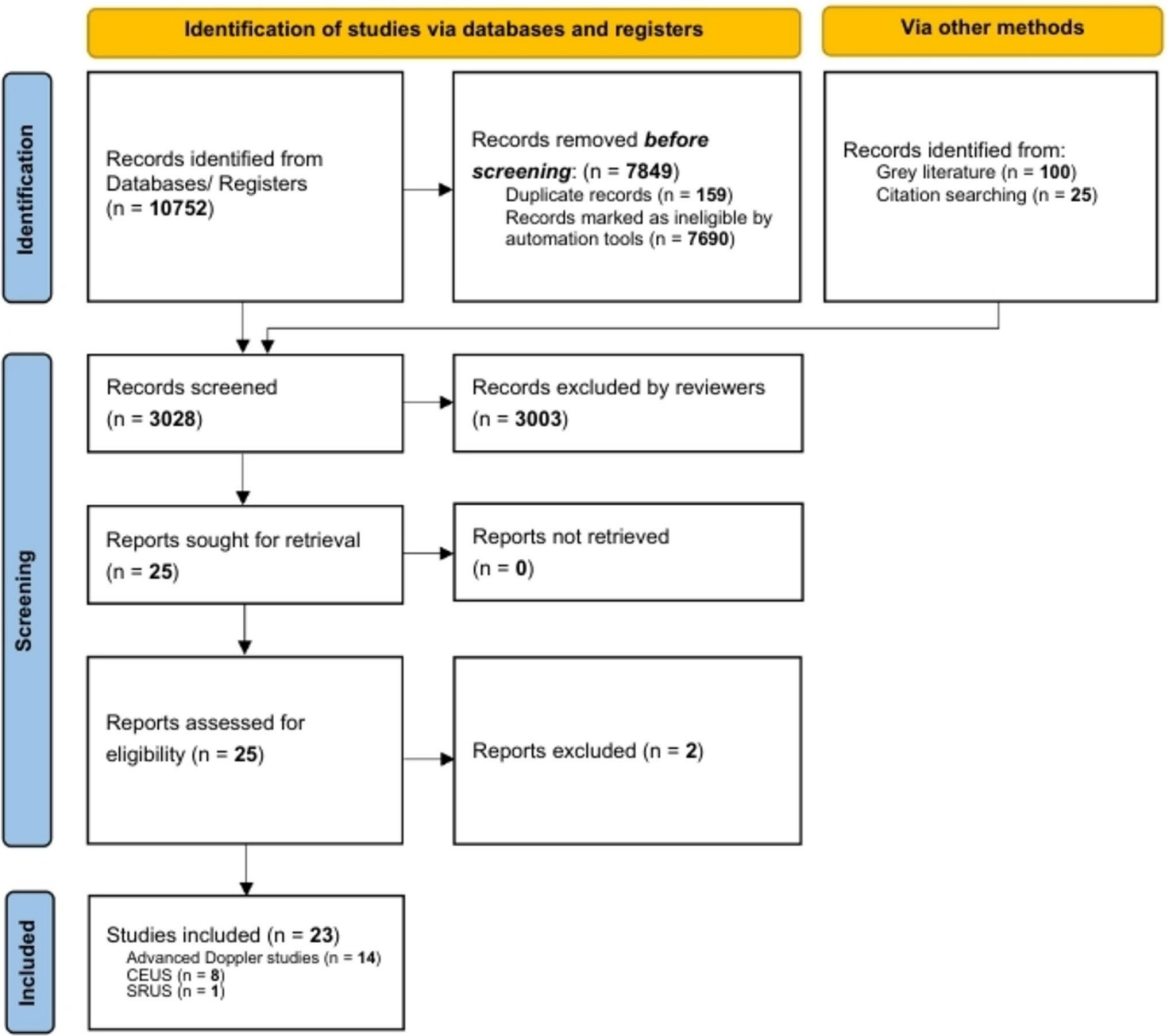
PRISMA diagram

We identified a total of 10,752 records from three databases, cross-referencing, and manual searches. After removing 159 duplicates, 7690 were assessed as ineligible. A total of 3028 records were screened, and 24 reports were assessed for eligibility using a full-text review. Ultimately, 23 studies (14 MVD, 8 CEUS, and 1 SRUS), including 354 infants from France, Germany, Austria, Italy, the USA, Canada, and China, were included in the final analysis (Fig. 1) [18, 24–44]. Amongst these, 163 brain CEUS scans were performed on 91 neonates born between 25^+3^ and 41^+4^ weeks of gestation, as shown in Table 1, with various US system parameters summarised in Table 2. Microvascular Doppler studies (*n* = 14) were identified through full-text review and investigated microvascular properties using ‘high frame-rate’ or ‘ultrafast’ Doppler microvascular flow imaging without contrast, as shown in Table 3. All contrast studies used the off-label second-generation microbubble (MB) ultrasound contrast agent SonoVue® (Bracco, Italy), known as Lumason® in the USA. A single study of 15 neonates produced 46 SRUS images using CEUS. For quantitative analysis, either a custom MATLAB (The MathWorks, Inc., USA) workflow or Vuebox® (Bracco, Italy) software was used. Doses of SonoVue® ranged from 0.03 to 0.08 mL/kg, with SRUS requiring the highest doses. There were no reports of adverse events directly attributed to the use of the ultrasound contrast agents.

**Table 1:** Study details and key results of CEUS and SRUS papers

Author (Year, country)	Title	Aims	*n*	GA	Weight (kg)	CEUS/SRUS	Timing design	Conditions	CEUS parameters	SRUS parameters	ROI	Key results
Schwarz et al. [31] (2025, Germany)	Ultrasound super-resolution imaging of neonatal cerebral vascular reorganization	Monitor macro- and microvascular changes during endovascular surgery for VAGM	15 VAGM = 7CoA = 8	37 1/7 (34 + 6/7–40 + 1/7)	3 (2.45–4.6)	CEUS + SRUS	3 time points (45)	VAGM, CoA	TIC, RT, FT, colour-coded maps	MB a.u., density, dispersity, tortuosity, directivity, flow velocity	Cortical grey and subcortical white matter, putamen	1. SRUS reveals VAGM is associated with increased RT and FT prior to treatment 2. Endovascular interventions reduce RT, FT, microvascular flow velocities, and dispersity measures post-treatment
Davis et al. [18] (2025, USA)	Review: Microvascular ultrasound imaging in the neonatal brain: from advanced Doppler to super-resolution	A review article with an original picture case report of an infant with HII	1	NR	NR	SRUS	Single	HII	NR	Density and velocity maps	Coronal plane: lenticulostriate, subependymal, and deep medullary vessels	1. SRUS offers enhanced visualisation of microvasculature compared to MVI. The quality of the SRUS image was impacted by motion artefact, high microbubble concentration, and low frame rate
Squires et al. [24] (2022, USA)	Feasibility and safety of contrast-enhanced ultrasound of the neonatal brain: a prospective study using MRI as the reference standard	Compare the diagnostic performance of CEUS with MRI as a reference	26	38 (25 + 3–41 + 2)	NR	CEUS	Single	GMH, VM, SDH, IPH, acute/chronic ischaemia, any abnormality	None	N/A	Global	1. CEUS was well tolerated with no significant alterations in vital signs during scans, including in infants with cardiac disease and shunts 2. CEUS exhibited sensitivities and specificities for acute/subacute ischaemia and chronic ischaemia ranging from 87.5% to 100%
Sridharan et al. [25] (2022, USA)	The wash-out of contrast-enhanced ultrasound for evaluation of hypoxic ischemic injury in neonates and infants: preliminary findings	Compare cerebral perfusion using the wash-out phase of the TIC curve in healthy and injured infants	6 healthy = 3HII = 3	35 (34–37)	NR	CEUS	Single	Healthy, HII	Wash-out metric ratios	N/A	Whole brain, cortex, cortical/subcortical grey and white matter. and central grey nuclei	1. Infants with hypoxic ischaemic injury are associated with a delayed wash-out rate (WoR) of contrast
Rüffer et al. [26] (2022, Germany)	Equal cerebral perfusion during extended aortic coarctation repair	Quantitatively evaluate cerebral perfusion during REEA using CEUS intraoperatively	9 CoA = 9	NR	3.1 (2.2–4.4)	CEUS	3 time points (27)	CoA, interrupted aortic arch type A	11* flow parameters	N/A	Both hemispheres	1. There is homogenous cerebral blood flow in both hemispheres during extended aortic coarctation repair with CPB
Knieling et al. [27] (2022, Germany)	Transfontanellar contrast-enhanced US for intraoperative imaging of cerebral perfusion during neonatal arterial switch operation	Use CEUS to quantify cerebral perfusion in neonates undergoing ASO	12 TGA = 12	39 + 5 (38 + 1–41 + 4)	3.6 (2.7–4.1)	CEUS	3 time points (36)	TGA	11* flow parameters	N/A	Both hemispheres	1. CEUS depicts a transient but significant reduction in cerebral perfusion during low-flow CPB and induced hypothermia 2. Neonatal age at surgery negatively correlated with flow, meaning that as age increased, flow measurements decreased (WiWoAUC)
Hwang et al. [28] (2019, USA)	Novel quantitative contrast-enhanced ultrasound detection of hypoxic ischemic injury in neonates and infants: pilot study 1	Can CEUS identify hypoxic ischaemic injury in at-risk infants)	8	36 (35–term)	NR	CEUS	Single	Healthy HII	PE, TTP, RT, WIR, WiAUC, WiPI. Ratios of CGNC	N/A	Cortex, central grey nuclei	1. All babies presented with abnormal neurology. 5 had evidence of HII on imaging 2. Central grey nuclei to cortex ratios of perfusion were altered in the setting of HII, with a higher CGN–cortex perfusion ratio 3. Both CEUS and MRI arterial spin labelling generate comparable perfusion maps
Hwang et al. [29] (2017, USA)	Novel contrast-enhanced ultrasound evaluation in neonatal hypoxic ischemic injury: clinical application and future directions	Case reports of CEUS in HII	2 HII = 2	35, 40	NR	CEUS	Single	HII	TTP, WI slope, PE, AUC, mTT, Half FT	N/A	Global	1. CEUS confers superior depiction of focal perfusion deficits relative to conventional greyscale US
Kastler et al. [30] (2014, France)	Transfontanellar contrast enhanced ultrasound in infants: Initial experience	Compare CEUS to standard US and MRI in various neonatal conditions	12	32 (26 + 6–41)	NR	CEUS	Single	Various neurological conditions	Qualitative	N/A	Global	1. TCEUS findings were accurate in 10 of 12 cases when compared to MRI. No perfusion abnormalities were suspected on standard US alone 2. When compared to MRI findings, CEUS showed 88.9% sensitivity, 66.6% specificity, 88.9% PPV, and 66.6% NPV

GA and weights reported as median (range)

Abbreviations: *GA*, gestational age at birth in weeks; *VAGM*, vein of Galen malformation; *CEUS*, contrast-enhanced ultrasound; *SRUS*, super-resolution ultrasound; *MI*, Mechanical Index; *NR*, not reported; *CPB*, cardiopulmonary bypass; *REEA*, resection with extended end-to-end anastomosis; *HII*, hypoxic ischaemic injury; *PE* (dB), peak enhancement; *PI* (WiAUC/RT), perfusion Index. *11 flow parameters: FT: fall time; mTT: mean-transit-time; PE: peak enhancement; RT: rise time; TTP: time to peak; WiAUC: wash-in-area-under-the-curve; WiPI: wash-in perfusion index; WiR: wash-in rate; WiWoAUC: wash-in-wash-out-area-under-the-curve; WoAUC: wash-out-area-under-the-curve; WoR: wash-out rate

**Table 2:** Study methods and CEUS system parameters

Author	Scanner	Probes	Contrast	Dose (mL/kg)	MI	Freq (MHz)	Frame rate (Hz)	Cine clip (s)	Software	Scanned by
Schwarz et al. [31]	GE Logiq E10s R3	C3—10	Sonovue Bracco	0.08	0.07	4	14	80–120	VueBox, Bracco; MATLAB, R2020a	Neonatologist
Davis et al. [18]	Phillips Epiq 7G	NR	Lumason Bracco	0.08	0.05	NR	NR	60	NR	NR
Squires et al. [24]	GE Logiq E9, E10	C3—10, L-9	Sulphur hexafluoride	0.03 (± 0.1 mL)	0.09–0.14	NR	NR	30	GE Scanner software	Radiologist
Sridharan et al. [25]	PhillipsEpiq	C5—1	Lumason Bracco	0.03	NR	NR	NR	120	MATLAB	Radiologist
Rüffer et al. [26]	Zonare ZS3, Mindray	C9—3	Sonovue Bracco	0.04	0.15	NR	NR	NR	VueBox, Bracco	Physician
Knieling et al. [27]	Zonare ZS3, Mindray	C9—3	Sonovue Bracco	0.04	0.15	NR	NR	NR	VueBox, Bracco	Physician
Hwang et al. [28]	Phillips Epiq, Aplio i800	C5—1, PSI-70BT	Lumason Bracco	0.03	0.06	1–7	NR	120	MATLAB	Radiologist
Hwang et al. [29]	Phillips Epiq	C5—1	Lumason Bracco	0.03	0.06	13	NR	120	QLAB on scanner	Sonographer
Kastler et al. [30]	Acuson Sequoia Siemens	L2, L5, L7.5	Sonovue Bracco	NR	NR	NR	8	20	PACS	Radiologist

Abbreviations: *NR*, not reported

**Table 3:** Advanced microvascular Doppler studies (*n* = 14)

Author (Year, country)	Title	Modality	*n*	Conditions/Treatments	Key relevant findings
He et al. [32] (2025, China)	Exploring the feasibility and clinical impact of ultrasound microvascular flow imaging in detecting brain injury in hyperbilirubinemia neonates	MV-Flow	85	Hyperbilirubinaemia (*n* = 51)	1. The affected group showed altered grey values in the basal ganglia region on standard US, and the MV-Flow technique revealed and quantified the microvascular structure of this region
Fakhari et al. [33] (2025, Canada)	Towards quantitative assessment of cerebrovascular autoregulation in human neonates using ultrafast ultrasound imaging	UUI	12	Cardiopulmonary bypass at deep (18–20 °C, *n* = 6) and mild (32–34 °C, *n* = 6) hypothermia	1. Negative association between MAP and arterial CBV in the mild group 2. Arterial CBV remained stable as MAP increased in the deep group
Davis et al. [18] (2025, USA)	Review: Microvascular ultrasound imaging in the neonatal brain: from advanced Doppler to super-resolution	MVI	2	1. Hydrocephalus 2. Preterm (27 + 6) with PDA	1. Engorgement of the leptomeningeal vessels in the hydrocephalus
Fakhari et al. [35] (2025, Canada)	Automated classification of cerebral arteries and veins in the neonate using ultrafast Doppler spectrogram	UUD	40	Healthy	1. A/V classification of slow arteriole and venular flow is possible with ultrafast normalised Doppler spectrogram (NDS)
Foran et al. [34] (2024, USA)	Microvascular imaging findings in infants with bacterial meningitis: a case series	MVI	3	Bacterial meningitis	1. Marked hyperperfusion and vascular flow engorgement are depicted in MVI in the initial stages of bacterial meningitis
Aguet et al. [36] (2023, Canada)	Impact of cardiopulmonary bypass on cerebrovascular autoregulation assessed by ultrafast ultrasound imaging	UUI	10	Hypoplastic left heart syndrome undergoing a Norwood operation (*n* = 5); healthy (*n* = 5)	1. Regional cortical and deep grey matter perfusion (CBV) decreased during neonatal cardiac surgery while in CPB
Fakhari et al. [37] (2023, Canada)	Longitudinal assessment of cerebral blood volume variation in human neonates using ultrafast power Doppler and diverging waves	UUD	5	Cardiopulmonary bypass	1. CBV exhibited significant variation during bypass: on average, + 20% in the mid-sagittal full sector, − 10.5% in the cortical regions and basal ganglia
Hwang et al. [38] (2022, USA)	Review: Current understanding and future potential applications of cerebral microvascular imaging in infants	MVI	11	PPHN on ECMO, hydrocephalus, bacterial meningitis, tumour, vascular malformation, stroke, HII, extreme prematurity (24 weeks)	1. MVI showed greater detail of microvasculature as compared to colour Doppler 2. Cortical vessels are short, parallel to each other, and perpendicular to the brain’s surface. Medullary vessels are fan­like on coronal views and straight on sagittal views. Striatal vessels are curvilinear on coronal sections
Tierradentro-Garcia et al. [39] (2022, USA)	Utility of cerebral microvascular imaging in infants undergoing ECMO	MVI	30	ECMO	1. MVI is feasible during neonatal ECMO with findings of lenticulostriate vessel tortuosity (26/30, 86.7%) in the basal ganglia and thalami, and increased peri-gyral flow (10/24, 41.7%) in the cortical area 4. Cortical white matter vascular engorgement was associated with poor outcome as defined by death, seizure, and/or abnormalities on MRI (p = 0.03)
Baranger et al. [40] (2021, France)	Bedside functional monitoring of the dynamic brain connectivity in human neonates	UUI, fUS, rs-FC	9	Prematurity (28 ± 2 weeks), 1 term newborn with a KCNQ2 mutation with burst suppression	1. fUS reveals the intra- and interhemispheric connectivity in a very preterm neonate 2. FC reveals a disconnection of thalamo-cortical networks for preterm neonates 3. FC reveals abnormal patterns in a newborn with burst suppression
Barletta et al. [41] (2021, Italy)	Cerebral superb microvascular imaging in preterm neonates: in vivo evaluation of thalamic, striatal, and extrastriatal angioarchitecture	SMI	15	Extremely (< 28 weeks), very (28–31 weeks), and moderate to late (32–37 weeks) preterm	1. Microvascular visibility increased with GA for superficial cortical and medullary vessels, while striatal and thalamic vessels were visible in all neonates, irrespective of their GA
Goeral et al. [42] (2018, Austria)	Microvessel ultrasound of neonatal brain parenchyma: feasibility, reproducibility, and normal imaging features by superb microvascular imaging (SMI)	SMI	19	Healthy-term newborns	SMI performance was better on coronal views than on sagittal views in visualising microvasculature (cortical, medullary, and striatal vessels)
Demene et al. [44] (2014, France)	Functional ultrasound imaging of brain activity in human newborns	UfD functional US	8	1. Sleep states at term (*n* = 6) 2. Neonatal seizures (*n* = 2)	1. UfD differentiates quiet and active sleep states. MV blood volume fluctuations are detected during seizures
Demené et al. [43] (2017, France)	Ultrafast Doppler reveals the mapping of cerebral vascular resistivity in neonates	UUD	14	Full-term (*n* = 9), very preterm (*n* = 5)	1. Microvascular resistivity changes during and after whole-body cooling 2. UUD enables quantitative mapping of deep-brain vascular dynamics and RI 3. 2D maps of resistivity index (RI) agree with measurements obtained on large arteries using conventional PW Doppler 3. Arterial and venous flow were differentiated based on distinct haemodynamics

Abbreviations: *MV-flow, *Microvascular flow, *ECMO, *extracorporeal membrane pulmonary oxygenation, *UUI,* ultrafast ultrasound imaging, *CBV,* Cerebral blood volume, *MVI, *Microvascular imaging, *CPB, *cardiopulmonary bypass, *PPHN,* persistent pulmonary hypertension, *HII, *Hypoxic ischaemic injury, *fUS, *Functional ultrasound, *rs-FC, *Resting state functional connectivity, *SMI, *Superb microvascular imaging, *PW, *Pulse wave

### Contrast-enhanced ultrasound

CEUS studies included healthy term and preterm infants, infants undergoing therapeutic hypothermia, and neonates with congenital cardiac disease or neurovascular conditions studied before, during, and after surgical or endovascular interventions. Doppler-based microvascular studies investigated physiological states (including sleep-state-dependent changes) and pathologies, including congenital cortical disease, hyperbilirubinaemia, and meningitis. Kastler et al. was the first study to show CEUS accuracy in detecting perfusion abnormalities in correlation with MRI imaging and reported that standard 2D US did not detect any changes [30]. CEUS revealed homogenous cerebral blood flow in cardiac surgery during cardio-pulmonary bypass (CPB) in infants with TGA and coarctation of the aorta (CoA) during extended aortic coarctation repair [26, 27]. There was a transient but significant reduction in cerebral perfusion during low-flow CPB and induced hypothermia. CEUS was well tolerated in a cohort of 26 neonates ranging from 25^+3^ to 41^+2^ weeks of gestation with no significant alterations in vital signs during scans [24]. In this cohort, CEUS alone was able to detect acute, subacute, and chronic ischaemia with sensitivities and specificities ranging from 87.5 to 100%. Knieling et al. reported that neonatal age at surgery negatively correlated with flow, meaning that as age increased, flow measurements decreased in pre-operative infants with TGA [27]. Interestingly, CEUS also revealed superior depiction of focal perfusion deficits relative to conventional greyscale US in hypoxic–ischaemic injury (HII) [29]. This group studied specific regions of interest (CGN, cortex, and whole brain) and assessed the perfusion pattern by measuring wash-in and wash-out parameters of CEUS. The majority of CEUS studies (6/9) performed a single scan, while three studies performed longitudinal scans at three time points, allowing for dynamic comparison of perfusion in the same infant. All these infants had diagnosed brain abnormalities or congenital heart disease, compared with healthy recruits.

### Super-resolution ultrasound

One neonatal proof-of-concept study applied ULM to CEUS acquisitions, achieving microvascular mapping at approximately 10 μm resolution and suggesting potential clinical utility in complex neurovascular disease such as vein of Galen malformations (VAGM), including peri-intervention assessment of microvascular reorganisation and flow velocity distribution changes [31]. They initially investigated CEUS perfusion parameters between 2 cohorts: 7 infants with VAGM and 8 infants with CoA. Scans were performed at 3 time points (pre-intervention, 24 h post-intervention, and 1 week post-intervention). Results show that a longer rise time (RT) and fall time (FT) seen in the time–intensity curves (TIC) prior to therapy is a hallmark of VAGM, and endovascular interventions decrease RT and FT by 24 h post-treatment. ULM was used to calculate absolute microbubble flow velocities and demonstrated that the smallest and slowest-flow vessels show a pronounced flow increment post-treatment, whereas the faster-flow vessels were not affected by flow changes during therapy. This flow acceleration was not accompanied by any changes in the anatomy of vessels in terms of dispersity or tortuosity. They reported one infant with a complication of left-sided parenchymal haemorrhage surrounded by an infarcted area, demonstrating a relative increase in MB speed and dispersity with a decrease in tortuosity and distance metric in the infarcted area.

### Microvascular Doppler

MVD techniques aim to improve the detection of slow flow in small vessels compared with conventional Doppler. Clinically available methods such as superb microvascular imaging (SMI) use advanced clutter suppression algorithms applied to conventional Doppler acquisition to enhance visualisation of small cortical and deep vessels [41, 42]. In contrast, ultrafast Doppler (UfD) employs high frame-rate plane-wave imaging, enabling highly sensitive detection of microvascular flow and dynamic cerebral activity mapping. Demene et al. provided proof-of-concept that UfD can map deep brain microvascular dynamics and differentiate between quiet and active sleep states [44]. They investigated 2 infants with congenital cortical disease and showed microvascular blood volume fluctuations (order of mm/s) during ictal activity detected on electroencephalogram (EEG). He et al. reported infants with hyperbilirubinaemia demonstrating a higher globus pallidus-to-putamen (G/P) ratio, and reduced MVD-measured vascular index, indicating lower cerebral microvascular flow in central grey matter with higher bilirubin levels [32]. Fakhari et al. showed variation in cerebral blood flow in specific brain regions in infants undergoing cardiopulmonary bypass and were able to classify slow arteriole and venular flow [33, 35, 37]. Foran et al. used MVI to show microvascular engorgement in early stages of bacterial meningitis [34].

## Discussion

**Fig. 2:**
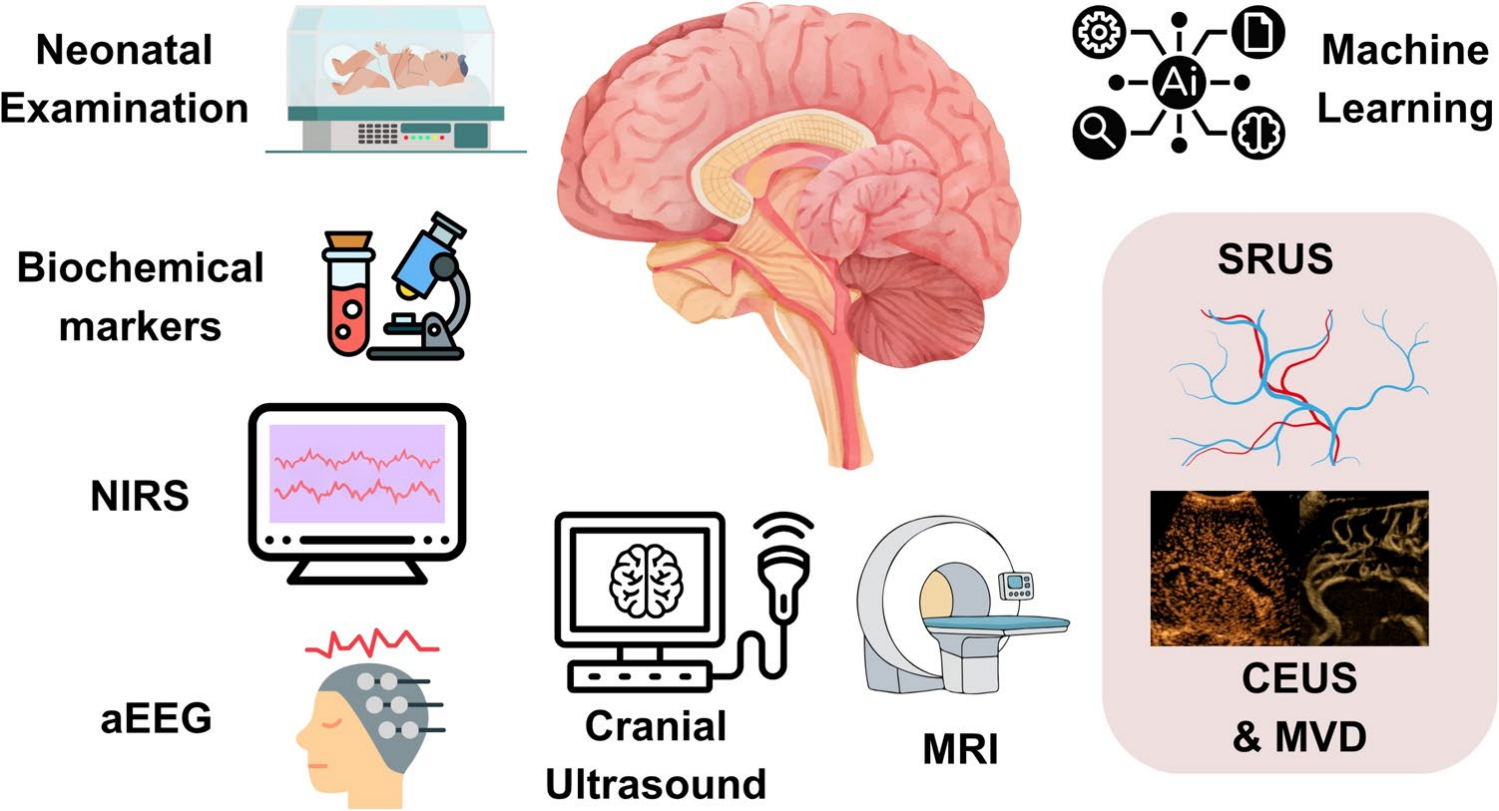
Methods of structural and functional assessment of the neonatal brain: (**a**) neonatal examination of neurological status (Hammersmith, NICHHD, modified Sarnat assessments), (**b**) routine 2-D cranial ultrasound scan, (**c**) near-infrared spectroscopy (NIRS) to measure cerebral regional tissue oxygenation, (**d**) amplitude-integrated EEG (aEEG); reliably used in late preterm and term neonates, (**e**) MRI (used at term-corrected age in preterm neonates and in term neonates, (**f**) biochemical markers such as serum lactate, LDH, and troponin, (**g**) microvascular imaging techniques such as contrast-enhanced ultrasound (CEUS), microvascular Doppler (MVD), super-resolution ultrasound (SRUS) scans, and (**h**) machine learning algorithms to combine all assessments for disease characterisation and stratification

This scoping review demonstrates that advanced microvascular ultrasound techniques can be used safely in neonates at the bedside, with early studies suggesting feasible acquisition protocols. Across the current literature, CEUS offers dynamic perfusion information and, when paired with SRUS/ULM processing, enables microvascular mapping beyond the diffraction limit. Ultrafast Doppler-based approaches offer a contrast-free microvascular signal and have demonstrated interesting physiological sensitivity but remain constrained by resolution limits. These techniques appear best as complementary to existing neonatal neuroimaging tools rather than replacements for standard cranial ultrasound or MRI (Fig. 2).

**Fig. 3:**
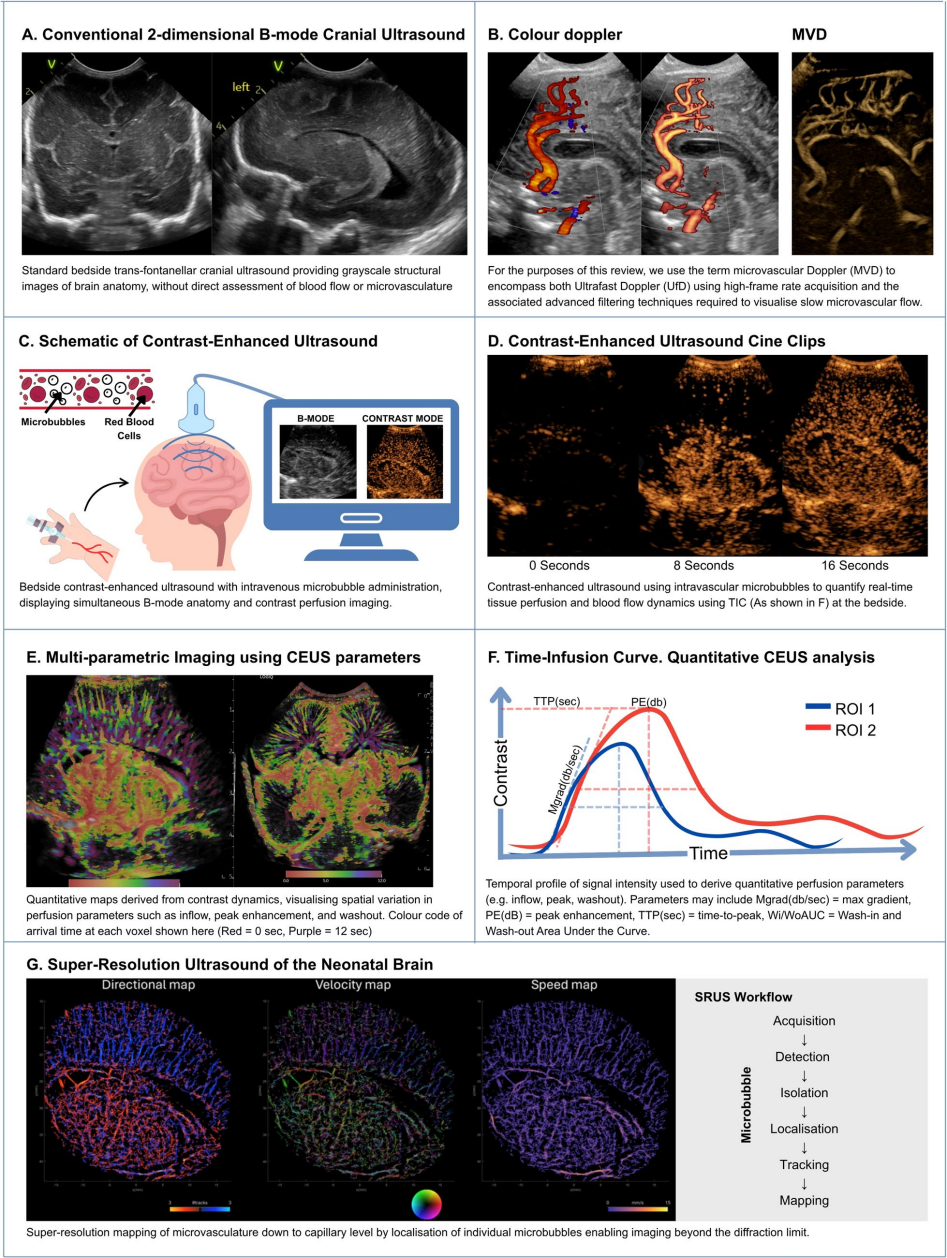
A schematic of cranial ultrasound workflow, including **A** routine 2D ultrasound, **B** colour Doppler and microvascular Doppler (MVD) imaging, **C** schematic diagram of contrast-enhanced ultrasound (CEUS) workflow, **D** CEUS still images at 0, 8, and 16 s during cine clip recording, **E** multi-parametric imaging using colour to demonstrate time of contrast arrival, **F** diagram of time-infusion curve (TIC) demonstrating the perfusion markers, and **G** super-resolution ultrasound (SRUS) imaging and ultrasound localisation microscopy (ULM) workflow

CEUS is currently the most accessible of these approaches, with modern ultrasound systems allowing contrast capability. CEUS distinctly interrogates whole-organ tissue perfusion at the microvascular level using region-of-interest approaches and quantitative time–intensity curve analysis (Fig. 3). Although not reported in the literature, CEUS acquisitions also enable multi-parametric imaging of the neonatal brain to generate complementary regional maps of quantitative perfusion metrics, thereby providing a richer assessment of cerebral perfusion than any single parameter alone. These perfusion parameters have been explored in basal ganglia and cortical regions relevant to HIE phenotypes and in perioperative contexts where cerebral perfusion may fluctuate rapidly. Importantly, CEUS is portable and repeatable, enabling longitudinal assessment during the precise window when injury evolves and when neuroprotective strategies are deployed. The safety profile of ultrasound contrast agents has been well described in neonatal populations [45–53]. Across published series, serious adverse events are rare, and haemodynamic instability has not been observed. In our own experience, CEUS was well tolerated, and no contrast-related adverse events were identified at the time of discharge in neonates, including extremely premature infants. These findings support the feasibility of conducting contrast-enhanced studies in a monitored neonatal intensive care environment.

CEUS offers a pragmatic advantage compared with MRI perfusion methods by employing microbubble contrast agents that remain strictly intravascular as true blood-pool agents. However, gadolinium-based MRI contrast agents are small molecules that may extravasate into the interstitial space when the blood–brain barrier is disrupted. Additionally, conventional non-contrast MRI vascular techniques such as time-of-flight imaging and arterial spin labelling provide macrovascular anatomical detail, or bulk perfusion estimates but cannot resolve capillary-level microvascular architecture. MRI remains the gold standard for tissue diagnosis and prognostication, but repeated early perfusion imaging is impractical in unstable neonates. CEUS can be integrated into bedside workflows and paired with physiologic monitoring (for example, NIRS and targeted haemodynamic assessment) to study how systemic interventions translate into cerebral microvascular perfusion responses in real time.

While CEUS yields perfusion metrics at the tissue level, SRUS/ULM adds a new dimension: structural and functional microvascular mapping beyond the diffraction limit. By localising individual microbubbles and reconstructing microvascular networks, ULM can resolve vessel geometry and flow velocity distributions at micrometre scales. Although neonatal evidence is limited, the first neonatal proof-of-concept work in complex neurovascular disease suggests that SRUS can detect microvascular reorganisation after interventions and quantify flow acceleration in the smallest vessels—signals that are not accessible to conventional Doppler and not fully captured by ROI perfusion averaging alone [31]. This creates an opportunity to develop mechanistic biomarkers (e.g., dispersity, tortuosity, distance metrics, percentile flow velocity shifts) that may align more directly with microvascular pathophysiology and recovery trajectories.

The available evidence remains early and methodologically heterogeneous. Most studies were small, single-centre, and exploratory, with variation in patient populations, imaging platforms, acquisition settings, contrast dosing, analytical pipelines, and reported outcome measures. This limits direct comparison between studies, and a formal risk-of-bias assessment was not undertaken. Rather than supporting a shift away from established bedside tools such as Doppler ultrasound, near-infrared spectroscopy, or MRI, the current evidence suggests that microvascular ultrasound techniques may add valuable complementary information, particularly where regional perfusion or microvascular dysfunction is central to pathophysiology.

### Clinical implications and future directions

From a clinical perspective, microvascular ultrasound techniques should be viewed as complementary to, rather than replacements for, standard cranial ultrasound and MRI. In practice, CEUS could provide repeatable, region-specific qualitative or quantitative perfusion assessment when conventional imaging using Doppler indices is difficult to interpret in the presence of shunts, ventilatory changes, vasoactive therapy, or evolving illness severity. This may be particularly relevant in infants with risk of IVH, suspected HII, haemodynamically significant PDA, congenital heart disease around surgery, and complex neurovascular lesions, where systemic haemodynamics can change rapidly, and microvascular perfusion may be the most distal marker of evolving cerebral vulnerability.

Translation into meaningful clinical tools will depend on standardisation and validation. Multicentre studies are needed to harmonise acquisition protocols (probe position, mechanical index, frame rate, contrast dosing, ROI definition), define gestation- and postnatal age-specific reference ranges, and establish reproducibility across operators and platforms. Critically, future work should prioritise longitudinal linkage between early perfusion/microvascular metrics and clinically important short and long-term neurodevelopmental outcomes, with transparent adjustment for confounders and prespecified analytic plans.

Finally, SRUS/ULM offers a research pathway to mechanistic biomarkers that could underpin the next generation of neonatal neurovascular phenotyping. By enabling microvascular network mapping and flow-velocity distributions beyond the diffraction limit, SRUS could help disentangle heterogeneous pathways to injury (e.g. capillary transit heterogeneity, venous congestion, reperfusion dynamics) and provide sensitive endpoints for early-phase trials of haemodynamic and neuroprotective interventions (Fig. 3)**.** Realising this potential will require careful governance given off-label contrast use in neonatal settings, robust safety reporting, and integration with existing bedside monitoring (e.g. NIRS, echocardiography, blood pressure targets) to evaluate whether microvascular ultrasound can improve decision-making and, ultimately, outcomes.

### Research agenda and translational priorities:

 To translate these techniques into clinically meaningful tools, we propose several priorities:

1. Standardisation:
  International consensus on acquisition protocols (probe position, mechanical index settings, frame rates, region of interest (ROI) definitions), contrast dosing strategies, and reporting standards are needed to reduce heterogeneity and enable pooled analyses.
2. Normative datasets:
  Just as the absence of robust normative trajectories in neonatology undermines interpretation of Doppler indices, CEUS and SRUS require gestation- and postnatal age-specific reference ranges for perfusion and microvascular metrics, including variability across sleep states and systemic haemodynamic conditions.
3. Longitudinal outcome linkage:
  Future studies must connect early microvascular metrics to clinically meaningful outcomes (MRI injury patterns, neurodevelopment, and functional outcomes), with appropriate adjustment for confounders and transparent reporting of missing data and reliability.
4. Safety governance and clinician training:
  Although published neonatal series report no severe adverse events, broader implementation will require standardised safety pathways, pharmacovigilance frameworks, and training analogous to other neonatal point-of-care ultrasound programmes. Clear governance is particularly important given the off-label nature of ultrasound contrast agent use in the neonatal population.
5. Comparative effectiveness against existing pathways:
  The critical question is whether CEUS/SRUS can help make decisions to modify outcomes: earlier identification of altered tissue perfusion states, improved targeting of haemodynamic interventions, and better prediction of evolving injury than current serial cranial ultrasound.

## Conclusion

Advanced microvascular ultrasound techniques such as MVD, CEUS, and SRUS represent an important development in neonatal neuroimaging, enabling bedside assessment of parenchymal perfusion and microvascular architecture and physiology at unprecedented resolution. Traditional arterial Doppler indices have shown limited reliability in predicting brain injury or long-term outcome in preterm infants, underscoring the need for approaches that interrogate microvascular physiology more directly. Neonatal CEUS and SRUS data, as shown here, demonstrate feasibility, tolerability, and potential clinical relevance across conditions including intraventricular haemorrhage, hypoxic–ischaemic injury, congenital heart disease, and neurovascular disorders. Given the accessibility of ultrasound, future progress will depend on standardised imaging protocols, the establishment of normative datasets, longitudinal outcome validation, and clear governance frameworks to support safe implementation. With coordinated multicentre collaboration, advanced microvascular ultrasound may become a valuable adjunct to existing neuroimaging pathways in neonatal care. This technology is ready, and the lifelong burden of prematurity and neonatal brain injury for babies, parents, healthcare systems, and our communities signifies an enormous potential for impact.

## Supplementary Information

Below is the link to the electronic supplementary material.

ESM 1 (DOCX 18.4 KB)

## Data Availability

No datasets were generated or analysed during the current study.
