# Supplementary material for: Microvascular ultrasound imaging of the neonatal brain: a scoping review
Source: Eur J Pediatr. 2026 May 21;185(6):422. doi: 10.1007/s00431-026-07042-x (PMC13190443; doi:10.1007/s00431-026-07042-x)
Supplement: Supplementary file 1 — (DOCX 18.4 KB) [file 431_2026_7042_MOESM1_ESM.docx]

**SUPPLEMENTARY MATERIALS**

**1. Supplementary Table 1. Data Extraction Items**

| # | Item | | Definition |
| --- | --- | --- | --- |
| 1 | Author | | Authors of study |
| 2 | Publication year | | Year of Publication |
| 3 | Title | | Title of study |
| 4 | Country | | Country where data for study was collected |
| 5 | Aims | | Main aims of the study |
| 6 | Size of study population | | Number of infants involved in study and categories |
| 7 | Gestational ages | | Median and range of gestational ages |
| 8 | Weights | | Median and range of weights |
| 9 | CEUS or SRUS or UfUS | | Investigation of CEUS or SRUS or UfUS |
| 11 | Timing design | | Single scan or repeated scans |
| 12 | Materials: | Scanner | Ultrasound system used |
| 13 |  | Probes | Ultrasound probes used |
| 14 |  | Contrast | Type of ultrasound contrast used |
| 15 |  | Dose | Dose of contrast used |
| 16 |  | MI | Mechanical Index used |
| 17 |  | Software | Software used |
| 18 | Scanned by | | Professional performing ultrasound scans |
| 19 | Conditions | | Neonatal conditions studied |
| 20 | CEUS Parameters | | CEUS parameters reported in text, tables or figures |
| 21 | SRUS parameters | | SRUS parameters reported in text, tables or figures |
| 22 | Regions of interest | | Cerebral regions of interest studied |
| 23 | Key results | | Key relevant results |
| 24 | Additional Information | | Any additional but relevant information not listed above |

Abbreviations: CEUS = Contrast-enhanced ultrasound, SRUS = Super-resolution Ultrasound, MI = Mechanical index.
